# Use of Hemospray in the treatment of patients with acute UGIB – short review

**Published:** 2013-06-25

**Authors:** RD Babiuc, M Purcarea, R Sadagurschi, L Negreanu

**Affiliations:** * 2nd Medical Clinic of Gastroenterology, University Emergency Hospital, Bucharest; ** 2nd Nephrology Department, “Carol Davila” Clinical Nephrology Hospital, Bucharest

**Keywords:** Hemospray, endoscopic hemostasis, inorganic powder, upper gastrointestinal bleeding

## Abstract

Gastrointestinal bleeding remains one of the most important emergencies in gastroenterology. It has been widely accepted that the first-line treatment for acute upper gastrointestinal bleeding, especially peptic ulcer bleeding, is endoscopic hemostasis. Several techniques are available to achieve hemostasis during endoscopy. However, some 5%–10% of the patients still experience recurrence of bleeding after initial hemostasis with combined endoscopic therapy including injection, thermal coagulation, or mechanical hemostasis. Endotherapy for upper gastrointestinal bleeding can be challenging. A simple and effective method of endoscopic hemostasis would have a significant impact on the treatment of active gastrointestinal bleeding. Hemospray (Cook Medical, Winston-Salem, North Carolina, USA) is a novel hemostatic agent for the treatment of upper gastrointestinal bleeding. Its efficacy has been shown in peptic ulcer bleeding, as well as in cancer-related gastrointestinal bleeding and in patients taking antithrombotic therapy. These initial reports are very promising, but they are limited by the small number of patients. Further studies are needed to confirm the efficacy of Hemospray in the management of upper gastrointestinal bleeding.

## Background

Gastrointestinal bleeding remains one of the most important emergencies in gastroenterology. Despite a decrease in mortality, the rate is still high, at 5%–10% in patients with peptic ulcer bleeding, about 15% in those with variceal hemorrhage, and 11%–14% in a population-based series all causes [**1**]. It has been widely accepted that the first-line treatment for acute upper gastrointestinal bleeding, especially peptic ulcer bleeding, is endoscopic hemostasis. It is currently recognized to be an effective procedure for the reduction of the rate of rebleeding, the need for surgery and mortality [**2**]. The most commonly used modalities are epinephrine injection, thermal coagulation using heater probe or monopolar probe and mechanical hemostasis using hemoclips, frequently applied as a combination therapy. They are highly effective, with overall success rates of 85%–95% in controlling hemorrhage. However, some 5%–10% of the patients still experience recurrence of bleeding after the initial hemostasis with combined endoscopic therapy [**3**]. Endotherapy for upper gastrointestinal bleeding can be challenging. Bleeding may occur from sites that are difficult to approach, such as the posterior duodenal wall or the upper region of the lesser gastric curvature, and this may make it hard to place hemoclips or apply adequate pressure with coagulation probes [**4**] or lesions can be large and actively bleeding, which makes it difficult to visualize and treat. In such cases, a higher level of technical expertise is often required. A simple and effective method of endoscopic hemostasis would have a significant impact on the treatment of active gastrointestinal bleeding [**3**]. An ideal endoscopic hemostasis device would be one that does not require a direct contact with the bleeding point and one that does not cause further tissue damage that may result in more severe bleeding [**3**].

 Hemospray (Cook Medical Inc., Winston-Salem, North Carolina, USA) is a novel powder licensed for endoscopic treatment, which has been used for many years on the battlefield to control bleeding, particularly from irregularly shaped and high-pressure arterial wounds. The Hemospray material is a proprietary inorganic powder. It contains no botanicals or proteins from humans or animals. The material works in two different ways: as a mechanical barrier and by absorption. When in contact with the bleeding site, the powder forms a barrier over the vessel wall, quickly stopping the bleeding, and secondly, the absorbent powder increases the local concentration of clotting factors and enhances clot formation. Because the Hemospray powder cannot be taken in by mucosal tissues, absorption and metabolism of the powder does not occur in the body, thereby eliminating the risk of systemic toxicity [**3**]. The delivery device consists of a syringe containing the Hemospray powder (21g per syringe), a delivery catheter that is inserted into the working channel of the endoscope, and an introducer handle with a built-in carbon dioxide canister to propel the Hemospray powder out of the catheter [**3**]. The efficacy of this novel nano powder hemostatic agent (TC-325) has been explored in several studies. Firstly, Giday et al. evaluated the usefulness of Hemospray in achieving hemostasis by inducing severe gastric arterial bleedings in pigs. They reported 100% initial cessation of bleeding with the use of Hemospray versus 0% in the control group, which did not receive any treatment. Hemospray was found to be eliminated from the stomach of animals within 48 hours with no complication associated with passage of the powder through the gastrointestinal tract [**3**]. Based on this promising animal work, a clinical study was conducted: Sung et al. used the Hemospray in 20 patients with active peptic ulcer bleeding and achieved immediate cessation of the bleeding in 95%; the single patient in whom Hemospray failed had a bleeding pseudoaneurysm. After 3 days, Hemospray was found to be eliminated from the stomach and duodenum in all patients. No adverse effects were noted [**4**]. Three possible hazards of using Hemospray could appear embolization, intestinal obstruction and allergic reaction to the powder. The risk of embolism is very low as the pressure of the carbon dioxide used to propel the Hemospray powder is unlikely to overcome the arterial blood pressure, and this would be necessary to enable the powder to enter the bloodstream. Gastrointestinal obstruction is another possible risk as the powder is sloughed from the gastrointestinal wall and passes into the small intestine [**3**]. None of these reactions was observed in either of the two studies. 

The fact that Hemospray covers a wide area of the gastrointestinal mucosa may also make it useful in treating generalized hemorrhagic lesions such as hemorrhagic gastritis, vascular ectasias (such as watermelon stomach or gastric antral vascular ectasia [GAVE]), and tumor bleeding [**3**]. A descriptive case series of five patients with malignancy-related upper gastrointestinal bleeding was conducted by Chen and evaluated the efficacy of Hemospray in achieving hemostasis. All patients suffered from advanced malignancy including gastric adenocarcinoma with bleeding from an antral mass, adenocarcinoma of the distal esophagus with contact bleeding, pancreatic adenocarcinoma with bleeding from duodenal ulceration, non-small cell lung cancer with bleeding from an ulcerated metastasis in the gastric cardia, and breast cancer with bleeding from the duodenal metastasis [**5**]. Upper endoscopy was performed in all five cases with 15-20 g of Hemospray applied to the tumor surface; initial hemostasis was achieved in all patients. Four patients had no further documented rebleeding after being followed up for 13-41 days. These promising results suggest that Hemospray has the potential to be the new standard of care for tumor-related bleeding given its ease of application to large surface areas, even in difficult positions, without inducing further mucosal damage [**5**]. This is an open door for further studies on this topic.

 A large proportion of patients with gastrointestinal bleeding are on a daily dose of antiplatelets and anticoagulants in order to prevent ischemic cardiac and neurologic events. Both classes of medication precipitate and make control of bleeding more difficult [**6**]. As a consequence, the treatment of such patients with the current endoscopic modalities may be a challenging task and novel approaches, such as Hemospray use, should be evaluated to overcome this [**7**]. There is only one study that evaluates the efficacy of this hemostatic powder in the treatment of upper gastrointestinal bleeding in patients taking antithrombotic therapy. Holster et al. present the outcomes of 16 patients treated with Hemospray (8 taking antithrombotic therapy for various indications and 8 not). Initial hemostasis was achieved after Hemospray application in 5/8 patients on antithrombotic therapy (63%) and in all eight patients not on therapy. Rebleeding rates were similar in both groups. Failed initial hemostasis and rebleeding in the antithrombotic therapy+ group was almost exclusively observed when Hemospray was applied to arterial spurting bleeds. These data suggest that endoscopic hemostasis by Hemospray is not hampered by the systemic antithrombotic effects. As Hemospray was also successfully applied as salvage therapy, this may even suggest beneficial effects on clot formation that cannot be achieved by current endoscopic hemostatic modalities such as clipping [**7**]. 

 What about that other night-disturbing cause of severe upper gastrointestinal bleeding: variceal bleeding? Will more or less blind spraying of the material into a blood-filled distal esophagus or gastric fundus stop these bleedings too? In variceal bleeding, the risk of the material entering the bloodstream, with the potential risk of embolus formation, might be higher than in arterial bleeding [**4**]. Currently, there is no study assessing this application of Hemospray. We found only an isolated case report of bleeding gastric varices, which were successfully treated with hemostatic powder [**8**].

## Conclusions

As a conclusion, the data about this new endoscopic hemostatic method are spectacular and hold the promise of a real breakthrough in the treatment of upper gastrointestinal bleeding. The technique appears surprisingly simple and should require a lower level of endoscopic skills than coagulation or clipping. However, there are some weaknesses in the studies discussed above. The main limitation is the small number of patients included, so larger prospective trials are needed in order to confirm the efficacy of Hemospray in daily practice. In the human study, only relatively mild bleedings were treated (95% had Forrest-type Ib lesions, i.e., oozing bleeding), and patients with hemodynamic instability were excluded [**4**]. The results of this study need to be validated in studies with larger sample sizes and in patients with actively bleeding peptic ulcers, and efficacy should be compared with other established endoscopic hemostatic therapies. 

 Nevertheless, these initial reports are very promising, and it is tempting to speculate about the potential role of this fascinating new treatment modality. Will Hemospray obviate the need for additional endoscopic treatment: will simply spraying the material on a spurting vessel suffice as a definitive therapy? Or will this technique be used as a temporary measure, stopping severe bleedings in an effective and simple manner? [**4**] One might also speculate that in severe arterial bleeding or in selected patients with variceal bleeding, Hemospray may act as a bridge towards surgery or TIPS insertion, respectively. 

 We eagerly await the results of further studies assessing the efficacy of Hemospray in patients with upper gastrointestinal bleeding, to see whether this Hemospray works as a “Magic Powder.”
